# Amphotericin B Encapsulation in Polymeric Nanoparticles: Toxicity Insights via Cells and Zebrafish Embryo Testing

**DOI:** 10.3390/pharmaceutics17010116

**Published:** 2025-01-16

**Authors:** Magno Maciel-Magalhães, Renata Jurema Medeiros, Nayara Cecília do Couto Guedes, Thais Morais de Brito, Gabriele Fátima de Souza, Beatriz Rodrigues Canabarro, Fausto Klabund Ferraris, Fábio Coelho Amendoeira, Helvécio Vinicius Antunes Rocha, Beatriz Ferreira de Carvalho Patricio, Isabella Fernandes Delgado

**Affiliations:** 1 Programa de Pós-graduação em Pesquisa Translacional em Fármacos e Medicamentos (PPG-PTFM), Fundação Oswaldo Cruz (Fiocruz), Rio de Janeiro 21040-900, Brazil; helvecio.rocha@fiocruz.br (H.V.A.R.); isabella.delgado@fiocruz.br (I.F.D.); 2 Programa de Pós-graduação em Vigilância Sanitária (PPG-VISA), Fundação Oswaldo Cruz (Fiocruz), Rio de Janeiro 21040-900, Brazil; thais.brito@fiocruz.br (T.M.d.B.); fausto.ferraris@fiocruz.br (F.K.F.); fabio.amendoeira@fiocruz.br (F.C.A.); 3 Departamento de Farmacologia e Toxicologia, Instituto Nacional de Controle de Qualidade em Saúde (INCQS), Fundação Oswaldo Cruz (Fiocruz), Rio de Janeiro 21040-900, Brazil; renata.medeiros@fiocruz.br (R.J.M.); gabriele.souza@fiocruz.br (G.F.d.S.); 4 Instituto Alberto Luiz Coimbra de Pós-Graduação e Pesquisa em Engenharia (COPPE), Universidade Federal do Rio de Janeiro (UFRJ), Rio de Janeiro 21941-594, Brazil; 5 Vice-Presidência de Produção e Inovação em Saúde (VPPIS), Fundação Oswaldo Cruz (Fiocruz), Rio de Janeiro 21040-900, Brazil; 6 Laboratório de Inovação Farmacêutica e Tecnológica, Departamento de Fisiologia, Instituto Biomédico, Universidade Federal do Estado do Rio de Janeiro (UNIRIO), Rio de Janeiro 24435-000, Brazil; 7 Vice-Presidência de Educação, Informação e Comunicação (VPEIC), Fundação Oswaldo Cruz (Fiocruz), Rio de Janeiro 21040-900, Brazil

**Keywords:** amphotericin B, polymeric nanoparticles, poly(lactic acid), polycaprolactone, zebrafish, toxicity

## Abstract

**Background:** Amphotericin B (AmB) is a commonly utilized antifungal agent, which is also recommended for the treatment of certain neglected tropical diseases, including leishmaniasis. However, its clinical application is constrained because of its poor oral bioavailability and adverse effects, prompting the investigation of alternative drug delivery systems. Polymeric nanoparticles (PNPs) have gained attention as a potential drug delivery vehicle, providing advantages such as sustained release and enhanced bioavailability, and could have potential as AmB carriers. However, concerns persist regarding nanomaterials’ toxicity, requiring more studies. Zebrafish (*Danio rerio*) embryos were used as a valuable model for toxicity testing, especially because of their genetic similarity to humans and standardized developmental assessments. **Methods:** In this study, we produced and characterized AmB loaded and non-loaded PNPs by nanoprecipitation, dynamic light scattering, transmission electron microscopy, atomic force microscopy and spectroscopy. Afterwards, we verified their toxicity through in vitro MTT assays in three cell lines (HEK293, HepG2, and J774 A1) and in vivo tests with zebrafish embryos. **Results:** In both trials, it was noted that nanoencapsulation of the drug led to increased toxicity when compared to non-encapsulated AmB, possibly indicating that they penetrated the embryo’s chorion. Nevertheless, it was demonstrated that the polymers used are safe and they are not the cause of toxicity, neither are the nanostructures per se. **Conclusions:** Therefore, it is believed that the objective of improving the bioavailability of AmB may have been achieved, and the observed toxicity was probably linked to AmB’s ability to destabilize cell membranes.

## 1. Introduction

Amphotericin B (AmB) is a polyene macrolide compound that has been employed since 1959 and continues to be utilized in the treatment of fungal infections. It was also explored for other medical conditions, such as neglected tropical diseases [1]. According to the biopharmaceutical classification system, AmB is categorized as a class IV drug as it exhibits poor permeability across the membranes of the gastrointestinal tract and low solubility in its fluids, defining it as a limited oral bioavailability drug [2]. Due to these limitations, AmB is primarily administered intravenously; however, various pharmaceutical formulations were developed, though they failed to bypass the need for intravenous delivery [3]. Recently, studies utilizing nanotechnology to address this challenge have increased significantly [4,5,6,7].

Polymeric nanoparticles (PNPs) have garnered considerable interest in the pharmaceutical sector in recent years due to their numerous advantages. These benefits include the possibilities of expanding the therapeutic window by minimizing toxicity, improving the drug’s bioavailability, and creating controlled release systems [8,9]. In recent years, some work was published involving the use of PNPs to encapsulate AmB, in an attempt to improve its bioavailability or achieve its targeted delivery, with most of them applying chitosan, a natural and biocompatible polymer [10]. Some biodegradable synthetic polymers were extensively studied in the development of medicinal PNPs, such as polycaprolactone (PCL), poly(lactic acid) (PLA), poly(glycolic acid) (PGA), and poly(lactic acid-co-glycolic acid) (PLGA). They were approved by the U.S. Food and Drug Administration (FDA) and the European Medicines Agency (EMA) due to their positive safety and biocompatibility profiles, low immunogenicity and toxicity, and good biodegradation in in vivo studies [11,12,13].

However, a critical point in the study of nanomaterials (NMs) is their toxicity compared to other scales. There is already a body of evidence indicating that the toxicity of NMs can exceed that of the same material produced on a micrometric scale [14,15]. A further challenge in the field of nanotoxicology is the wide range of chemical compositions, sizes, and conformations, factors that can affect the toxicity of NMs [15,16]. Currently, there are no toxicity tests that were developed especially for NMs, and those currently used, especially in vitro, have a series of limitations, including the ability to suspend NMs in cell culture media. Moreover, data available in the literature is still limited, making it hard to generalize about the risks posed by NMs to human health [16].

The zebrafish (*Danio rerio*) has become an animal model of wide importance in scientific research in recent years, especially for being a vertebrate animal with genetic similarity to humans with a high degree of gene homology (about 70%) [17]. The scientific literature describes its anatomy and physiological parameters, its liver, cardiovascular, and immune systems, which also share many significant physiological homologies with their mammalian counterparts [18]. Additionally, it is worth mentioning that since its first edition, the ISO/TR 16197:2014 standard has already established the testing in zebrafish embryos as a relevant in vivo model for studying nanomaterials’ toxicity. This is due to the rapid responses exhibited by the zebrafish embryo, which facilitate the expeditious generation of knowledge regarding the biological consequences of nanomaterial exposure [19].

Originally conceived as an alternative to the acute toxicity test in adult fish, the fish embryo toxicity test was internationally adopted as the OECD Test Guideline (TG) 236 in 2013 [20]. According to this TG, after mating, the test should be started as soon as possible, before the 16-cell stage of embryo development, and it is finalized at 96 h post-fertilization (hpf), looking for possible morphological changes due to toxicity generated by the substance being tested [20]. However, it can also be carried out up to 120 hpf if the researcher wishes to ascertain any anomalies in fish development [21]. Nevertheless, the severity of non-lethal malformations is subjective. Thus, to improve the comparability of these results, different research groups have used semi-quantitative scoring systems to evaluate the observed malformations and classify sample toxicity [22].

The present study aimed to carry out a series of toxicological tests of polycaprolactone (PCL) and poly(lactic acid) (PLA) PNPs containing AmB, their non-loaded correlates, and the free drug, applying the MTT assay with three cell types and the zebrafish embryo model, to verify whether changes in the drug’s toxicity would occur when nanoformulated.

## 2. Materials and Methods

### 2.1. PNP Production

PNPs were produced through a nanoprecipitation method [23]. Specifically, 80 mg of the polymer (PLA, CAS No. 26780-50-7, Mw 18,000–28,000, Sigma-Aldrich, St. Louis, MO, USA or PCL, CAS No. 24980-41-4, Mw 14,000, Sigma-Aldrich) was dissolved in 15 mL of acetone (CAS No. 67-64-1, Biograde, Nanjing, China), which was acidified with 150 μL of 0.1 N HCl (CAS No. 7647-01-0, Biograde). For formulations containing amphotericin B (AmB, CAS No. 1397-89-3, Hangzhou Dayangchem, Hangzhou, China), 20 mg of the drug was solubilized in 2 mL of dimethyl sulfoxide (DMSO, CAS No. 67-68-5, Merck, Rahway, NJ, USA), followed by the addition of 5 mL of methanol (CAS No. 67-56-1, Merck). Once both solutions became clear, they were combined to form the organic phase (OP). The aqueous phase (AP) consisted of 60 mL of a 0.3% (*w*/*v*) super refined polysorbate 80 (P80, CAS No. 9005-65-6, Croda, Snaith, UK) solution in ultrapure water, maintained at room temperature. The two phases were subsequently mixed using a burette with the tap opened, ensuring the continuous flow of OP, while AP was kept under constant stirring at 500 rpm using a magnetic stir plate. The mixture was then subjected to magnetic stirring for an additional 10 min at the same velocity. Following this, volatile organic solvents were eliminated using a rotary evaporator (IKA, RV8, Burladingen, Germany) set to 38 °C. Any excess AmB was removed from the PNP suspension via centrifugation (Thermo Fisher Scientific, Megafuge 8, Waltham, MA, USA) during a one-hour cycle at 985× *g* and room temperature. Initially, the nanoparticles utilized in this study were prepared in our laboratory without a subsequent ultracentrifugation step. However, based on the results obtained from the initial zebrafish embryo–larval acute toxicity test (ZELT) (Supplementary Data), two ultracentrifugation steps of 15 min each were introduced into the process, conducted at 20,000× *g* and 10 °C (Thermo Fisher Scientific, Sorvall™ MTX150, USA), with the solvent (ultrapure water) being changed at each round of ultracentrifugation in order to remove both P80 and residual DMSO. Finally, the precipitated nanoparticles were resuspended in ultrapure water and characterized.

### 2.2. Characterization of Produced PNP

To quantify the nanoencapsulated AmB, 500 μL of the PNP-AmB suspensions were transferred to microtubes containing a 100 kDa Amicon filter (Merck-Millipore, Molsheim, France). Centrifugation was conducted at 7500× *g* for 20 min at room temperature (Thermo Fisher Scientific, Megafuge 8, USA). The PNP was subsequently recovered from the filter using 200 μL of a mixture of acetonitrile (ACN) and dimethyl sulfoxide (DMSO) in a 6:4 ratio, and then diluted in a volumetric flask. Absorbance readings were taken using a spectrophotometer (Shimadzu UV 1800, Kyoto, Japan) at a wavelength of 411 nm, and the necessary calculations were performed to determine the concentration.

The hydrodynamic diameter, polydispersity index (PdI), and zeta potential of the PNPs were assessed using dynamic light scattering (DLS) with a He-Ne laser (λ = 633 nm) and a 90° fixed-angle detector (Malvern Zetasizer Nano ZS90, Worcestershire, UK). For these measurements, embryonic medium (E3 medium: 5 mM NaCl; 0.17 mM KCl; 0.33 mM CaCl_2_; 0.33 mM MgSO_4_) was employed as a dispersant, and readings were conducted in triplicate, comprising 15 runs of 10 s each.

To evaluate the shape and size of the PNPs, samples were analyzed by transmission electron microscopy (TEM) and Atomic Force Microscopy (AFM).

TEM: The samples were diluted in ultrapure water to achieve a concentration of approximately 50 ng/mL of AmB and deposited onto 200 mesh Lacey Formvar/Carbon copper grids (Ted Pella, Redding, CA, USA). The samples were then counterstained with phosphotungstic acid and analyzed in a transmission electron microscope (JEOL, JEM-1011, Tokyo, Japan) operating at an accelerating voltage of 80 kV.

AFM: The samples were diluted in water in the proportion of 1:10. An aliquot (~40 µL) of the sample was applied onto mica and dried under an N_2_ (g) atmosphere for 30 min. The samples were analyzed in an atomic force microscope (Bruker, Dimension Icon, Camarillo, CA, USA) located at CENABIO-UFRJ. Images were acquired in PeakForce QNM^®^ mode at room temperature using a cantilever with a nominal spring constant of 3 N/m, a nominal resonance frequency of 75 kHz, and a length of 225 μm. Images were obtained with a 512 × 512 pixel resolution and processed using the NanoScope Analysis 1.7 software (Bruker).

Entrapment efficiency (EE) and drug loading (DL) were determined by using the following equations:

EE(%)=WAmBF/WAmBI×100

DL(%)=WAmBF/WPol×100

where WAmBF: The amount of AmB calculated in final sample; WAmBI: the amount of AmB added to the preparation; WPol: the amount of polymer added to prepare the PNP.

### 2.3. Cell Viability Assessment by MTT Assay

The cytotoxicity assays were performed in human embryonic kidney cell line HEK293, human liver cancer cell line HepG2, and murine macrophage cell line J774A.1 by the MTT assay. The cells were cultured in DMEM medium (Sigma-Aldrich: D5648-1L) supplemented with 10% fetal bovine serum [FBS (Sigma-Aldrich: F9665)], 2 mM glutamine, 100 μg/mL streptomycin, and 100 U/mL penicillin (Gibico, Frederick, MD, USA: 0550). The cells were maintained in an incubator (Sanyo Scientific, Gunma-ken, Japan/COM-17AC) with 5% CO_2_ at 37 °C.

To assess cell viability, cells from the described lineages were centrifuged (Nova Técnica—NT 812) for 8 min at 1500 rpm and resuspended in 1 mL of culture medium for counting in a Neubauer chamber, followed by an adjustment of suspension concentrations to 1 × 105 cells/mL. In a 96-well microplate, the cells were incubated (100 µL/well) with 100 μL of the AmB-loaded PNP or the free drug (FD) samples in quadruplicate at 0.001, 0.01, 0.1, 1, or 10 µg/mL for 24 h. AmB FD was firstly solubilized in DMSO and then diluted in purified water. PNPs were directly diluted in purified water. After this period, 22 μL of MTT 5 mg/mL (thiazolyl blue tetrazolium bromide—Invitrogen, Carlsbad, CA, USA: M6494) were added to each well, and the plate was again incubated for 4 h. The absorbance intensity was measured in a Bio Tek microplate reader at 550 nm, with values processed by the SoftMax Pro 5.3 program.

### 2.4. Zebrafish Maintenance

AB wild-type zebrafish (*Danio rerio*) were maintained in an automated water recirculation system (Tecniplast ZebTEC ActiveBlue^®^, Buguggiate, Italy) with the following system water parameters: temperature 26 ± 1 °C; pH 7.5 ± 0.5; conductivity (500 ± 100 mS) × 100. Dry feed (Gemma) was provided once in the morning, and live feed (*Artemia nauplii*) once in the afternoon. The system was maintained in a room with an automatic light–dark cycle of 14/10 h. Animals used in this work were kept free of specific pathogens: *Pseudoloma neutrophilia*, *Aeromonas hydrophila*, *Pleistophora hyphessobryconis*, *Edwardsiella ictaluri*, *Pseudocapillaria tomentosa*, *Ichthyophthirius multifilis*, and *Mycobacterium* spp. The maintenance and mating of the zebrafish are procedures authorized by Fiocruz’s Animal Ethics Committee (CEUA/Fiocruz) under license number LW-38/19 and LW-29/23.

### 2.5. Zebrafish Embryo–Larval Acute Toxicity Test (ZELT)

ZELT was performed based on OECD TG 236 [24], with minor modifications, and all experiments were carried out in triplicates. The nanosuspensions were diluted to the desired concentrations in the E3 medium. For each concentration, 20 fertilized embryos were selected and individually distributed in 20 wells of 24-well plates with 2.0 mL of diluted suspension. The remaining four wells of the plate were destined for internal negative control, performed with pure E3 medium. A negative control 24-well plate was also prepared with an E3 medium in which the maximum mortality rate allowed was 10%. The studied plates were kept at 26 °C in a calibrated and verified lab oven (MMM Group, Ecocell, Monroe, WA, USA).

Firstly, the PNP containing AmB’s concentrations to be tested (4, 8, 16, 32, and 64 µg/mL) were estimated from a half maximum inhibitory concentration (IC_50_) previously obtained by the group in an in vitro cytotoxicity test (data not yet published), where this value was selected as the “average value” of the scale, and two concentrations above and two below were added. The same dilutions were performed for non-loaded PNPs, following the corresponding concentrations.

After the completion of the first round of experiments, the in vitro assays were repeated as described in Section 2.3. Using the newly calculated IC_50_ value, a new range of AmB concentrations was tested in ZELT (0.05, 0.1, 0.2, 0.5, and 1 µg/mL), both for AmB-loaded and non-loaded PNPs, as well as for AmB FD. In this second round, the experiments with the non-loaded PNPs were not repeated, since they had already proven safe in the first round, and there was no need to use more animals with them. However, it was necessary to test the solvent used to solubilize AmB. Thus, the 0.1% DMSO group was added. It is important to clarify that all AmB FD concentrations were prepared in a final solution of 0.1% DMSO.

Testing started at 1, 6, and 24 hpf and embryo development was followed at 24 h intervals until 120 hpf. The four test endpoints determined in TG 236 were investigated: (1) embryo coagulation; (2) the absence of heartbeats; (3) the lack of somites; and (4) failure on tail detachment.

After this period, the live animals were observed for other possible sublethal signs of toxicity, such as edema development and other body deformities, and recorded. The animals were contained in a drop of 15% low-density sodium carboxymethylcellulose (Sigma-Aldrich) over a microscope slide to be photographed and filmed for 15 s. Monitoring, image acquisition, and posterior studies were conducted in a Leica stereoscope, model S9i, operating with LAS X 3.7.4 software (Chemnitz, Germany).

After image evaluation, larvae were evaluated using a semi-quantitative scoring system to analyze morphological defects and survival. For this, a scoring scale ranging from 0 to 4 was constructed, based on the one used by Patricio (2016) [25], where zero means no apparent morphological alteration; 1 represents a single non-lethal morphological change; 2 symbolizes represent two non-lethal morphological alterations; 3 corresponds to more than 2 non-lethal morphological alterations; and 4 means death or occurrence of the alterations determined in OECD TG 236. Additionally, measurements of larvae body size (head-tail) and eye diameter were also performed. The heartbeats were determined using the videos obtained and a manual counter. Then, the counted values were extrapolated to 60 s thus obtaining beats per minute (bpm).

Finally, to verify the internalization of PNP + AmB and AmB FD in 120 hpf zebrafish larvae, a fluorescence microscope (Nikon, TS100-F, Kanagawa, Japan) was used, with the FITC filter (excitation 475 nm; emission 530 nm) applied.

### 2.6. Statistical Analysis

Statistical analyses were performed with GraphPad Prism 8.3.1 for MacOS. All data were evaluated for normality using the Kolmogorov–Smirnov test, and values >0.05 were considered normal. Subsequently, for the data that presented normal distribution, a one-way analysis of variance (ANOVA) was applied, the result being considered statistically different from the control group when *p* < 0.05, followed by Dunnett’s multiple comparison test. For the data with non-normal distribution, the Kruskal–Wallis test was applied, the result being considered statistically different from the control group when *p* < 0.05, followed by Dunn’s multiple comparison test.

## 3. Results and Discussion

### 3.1. PNP Characterization

**Figure 1:**
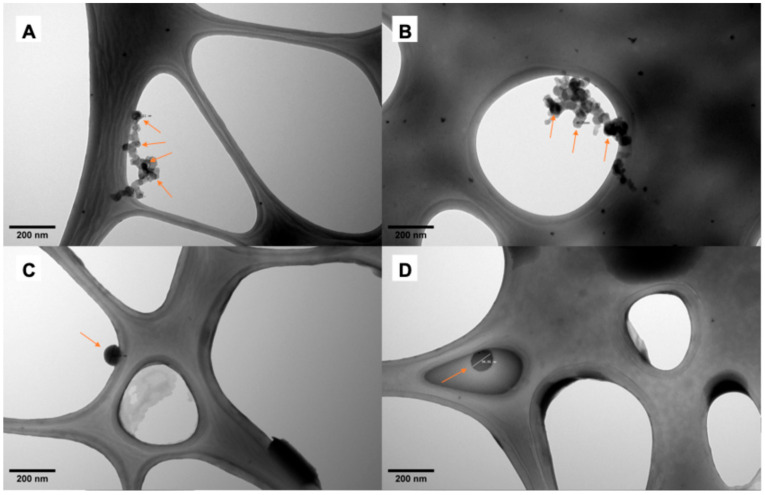
Transmission electron microscopy images of polymeric nanoparticles. (**A**) Non-loaded nanoparticles of poly(lactic acid); (**B**) nanoparticles of poly(lactic acid) loaded with amphotericin B; (**C**) non-loaded nanoparticles of polycaprolactone; (**D**) nanoparticles of polycaprolactone loaded with amphotericin B. Arrows are pointing at nanoparticles adhered to Lacey grid.

**Figure 2:**
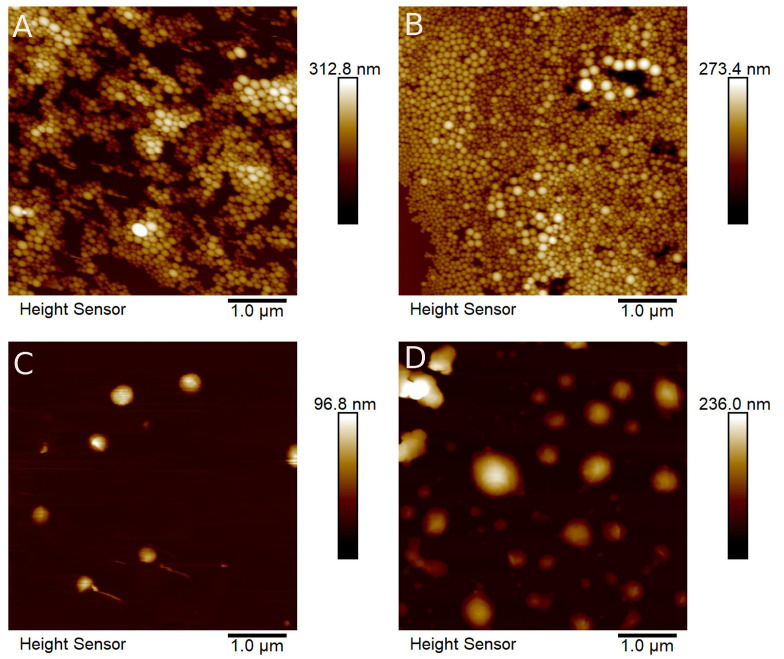
Atomic Force Microscopy images of polymeric nanoparticles. (**A**) Non-loaded nanoparticles of poly(lactic acid); (**B**) nanoparticles of poly(lactic acid) loaded with amphotericin B; (**C**) non-loaded nanoparticles of polycaprolactone; (**D**) nanoparticles of polycaprolactone loaded with amphotericin B.

PNP mean diameters were determined from the obtained TEM and AFM images (Figure 1 and Figure 2): poly(lactic acid) non-loaded nanoparticles (PLA NL) 25.50 ± 3.19 nm; poly(lactic acid) nanoparticles loaded with AmB (PLA + AmB) 40.11 ± 9.45 nm; polycaprolactone non-loaded nanoparticles (PCL NL) 84.82 ± 13.65 nm; and polycaprolactone nanoparticles loaded with AmB (PCL + AmB) 118.01 ± 19.07 nm. Their hydrodynamic diameters, PdI, and Zeta potential were also measured by the DLS technique, and the results are shown in Table 1.

As seen in the three techniques, PCL nanoparticles presented a tendency to be bigger in size than the PLA ones, and there was an increase in diameter after AmB encapsulation, suggesting it occurred successfully. Knowing that a PdI closer to 1 indicates a heterogeneous sample with different particle size populations [26,27], it is suggested that all samples have homogeneous particle size distributions (values found are between 0.018 and 0.142, closer to zero).

It was shown that, for colloidal systems with zeta potential values between 30 mV and −30 mV, attractive forces usually prevail over repulsive ones, causing particle aggregation [28]. This was not observed in the zeta potential measurements presented here and, leading us to believe that, in the E3 medium that is used for the ZELT test, the PNP did not aggregate. This alteration in the zeta potential may be related to the presence of the non-ionic surfactant (P80) adsorbed on PNPs’ surfaces, even though its excess has been removed. This reagent leads to a decrease in the electrophoretic mobility, affecting the measurement of the zeta potential, but without causing PNP aggregation [29].

EE and DL are important variables regarding the preparation of PNP + AmB since they measure the amount of drug internalized in them. Both were calculated using the mathematical formulas described in Section 2. The values obtained were relatively low, indicating that our preparation process would benefit from prospect improvements, leading us to consider other methods for the future, such as the one proposed by Saqib et al. (2020) [30], who also prepared PNP from PCL + AmB and obtained higher values. Nevertheless, as a limitation of the present study, it is worth mentioning the absence of drug release experiments, which were not possible to perform at the moment.

**Figure 3:**
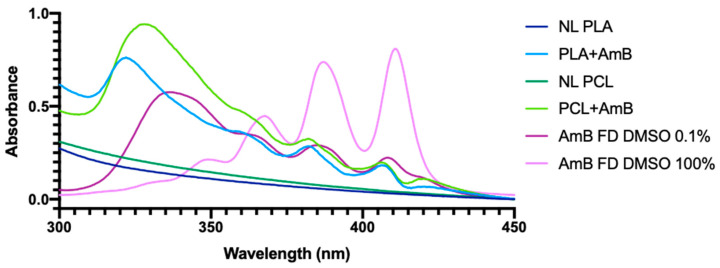
UV-Visible scanning spectra of NL PNP, PNP + AmB, and AmB FD samples. NL: non-loaded; PLA: poly(lactic acid); PCL: polycaprolactone; AmB: amphotericin B; FD: free drug; DMSO: dimethylsulfoxide.

In order to determine the agglomeration state of AmB in each sample, we used UV-Visible spectrum scanning, obtaining the spectra shown in Figure 3.

Regarding PNP + AmB, a main peak was observed between 322 and 328 nm, representing a hypsochromic change in relation to that observed in the AmB FD dissolved in DMSO 0.1%, whose prominent peak was located at 337 nm. The same was detected by Fernández-García et al. in a study where the researchers developed three different states of AmB agglomeration: monomeric, oligomeric, and poly-aggregated. In this study, the oligomeric state presented this 320–330 peak [31], which leads us to suggest that, in the case of our PNP + AmB, the process of PNP preparation also generated such a state. In the case of the AmB FD sample dissolved in DMSO 100%, the prominent peak at approximately 410 nm indicates the monomeric state of the drug [32].

### 3.2. MTT Assays

**Figure 4:**
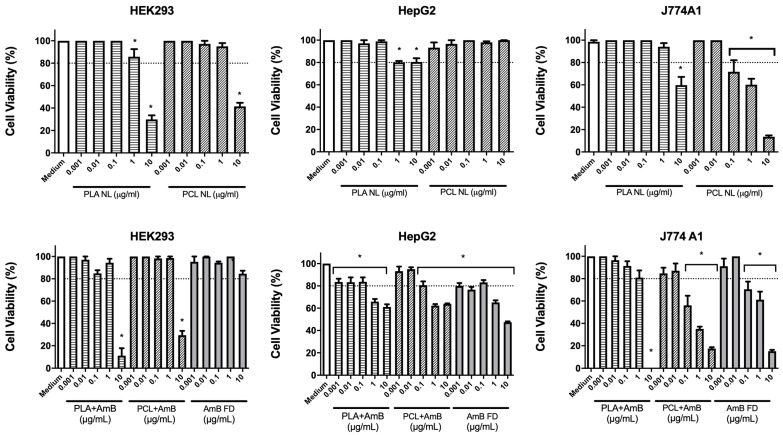
MTT assay for assessment of cell viability, using HEK293, HepG2, and J774.A1 cell lines, tested with non-loaded PNP, AmB-loaded PNP, or FD. AmB: amphotericin B; PCL: polycaprolactone; PLA: poly(lactic acid); NL: non-loaded; FD: free drug; PNP, polymeric nanoparticle. Data shown as mean ± standard deviation. * represents difference between sample and negative control (cell culture medium), with *p* > 0.05 in one-way ANOVA test followed by Dunnet’s multiple comparisons test. Dotted line marks the 80% threshold of cell viability.

In the cytotoxicity analysis, PLA NL, PCL NL, PLA + AmB, PCL + AmB, and AmB FD (0.001, 0.01, 0.1, 1, and 10 µg/mL) were tested across three cell lines. Results are shown in Table 2 and Figure 4, and it is noticeable that, in the J774.A1 cell line, PLA + AmB at the highest concentration resulted in 100% cell death, with no cytotoxic effects observed at the other concentrations. Likewise, in the case of PLA NL, a cytotoxic effect was only observed at a concentration of 10 µg/mL. However, cell death was not as pronounced, and it was possible to observe a difference between PLA NL and PLA + AmB in relation to their IC_50_. PCL NL, PCL + AmB, and AmB FD did not exhibit cytotoxic effects only at the two lowest concentrations; however, at 0.01, 1, and 10 µg/mL, a viability of less than 80% was observed for both treatments in a concentration-dependent manner.

Concerning the HepG2 cell line, both loaded PNPs exhibited a cytotoxic profile very akin to that of AmB FD, with less than 80% cell viability at the two highest concentrations. Nevertheless, for PNP NL, the results did not indicate prominent cytotoxicity at the concentrations tested, with viability above 80% for all cases. It is important to mention that, despite the reduction in cell viability mentioned for PNP + AmB, all of them obtained a calculated IC_50_ greater than 10 µg/mL, suggesting the non-occurrence of severe hepatotoxicity.

Regarding HEK293, both PLA + AmB and PCL + AmB exhibited high cytotoxicity at the highest concentration compared to the control group. However, the same effect was observed for the NL PNP at the same concentration, leading to a low variance between the IC_50_ calculated for these in comparison to their versions loaded with AmB. In contrast, AmB FD demonstrated over 80% viability at all tested concentrations in this cell line, and the highest IC_50_ among all samples.

HEK293 are human embryonic kidney cells, which are the main target organ of amphotericin B toxicity [33,34] and the reason for including them in the study. HepG2 is a human liver tumor cell line that is used both to study the metabolic functions of the liver and to verify the hepatotoxicity of substances, in this case, our AmB-containing nanoparticles. Since the liver is one of the organs most affected by toxicity in general [34], we decided to include this line. J774A.1 cells are murine macrophages, and applied in the present study to investigate the immunotoxicity of AmB-containing nanoparticles [35].

The data observed in this study, regarding the HEK293 cell line, suggests that the nanoencapsulation of AmB may have increased its permeability into the cells, rendering it more cytotoxic than AmB FD. The absence of toxicity from AmB FD is further corroborated by the work of Liu et al. (2020) [36], where a cytotoxic effect of AmB was observed starting at 12.5 µg/mL in the HEK293T cell line after 6 h of exposure.

The nephrotoxicity associated with AmB FD is common and dose-dependent. This occurs due to the accumulation of the drug in the renal tubules, as the kidneys are not only a crucial excretory organ but also receive approximately 25% of the cardiac output to sustain renal blood flow. Through the activation of the tubuloglomerular feedback system, AmB FD induces a reduction in renal blood flow alongside vasoconstriction of the afferent arteriole [37,38,39]. Consequently, ischemia and hypoxia occur in both tubular epithelium and endothelial cells, triggering the production of inflammatory mediators and reactive oxygen species (ROS). Ultimately, these ROS led to proteins and DNA oxidation as well as lipid peroxidation of cell membrane, resulting in oxidative damage followed by cell death [37,39,40,41].

However, this process occurs in living organisms thus involving interactions between multiple systems and organs, and perhaps this is the reason for the absence of toxicity of AmB FD in the human embryonic kidney cell line—HEK293—used in this study. Furthermore, the initial half-life of injectable AmB FD, which ensures 100% drug bioavailability, is 24–48 h [42] with the possibility of extending up to 15 days, while the exposure time for the HEK 293 cells was only 24 h.

Regarding the toxicity experienced by the J774A.1 cell line, our results for PNP + AmB can be compared to those obtained by Gedda et al. in a 2020 study [35], where the researchers prepared three amine functionalized carbon-based composite nanoparticles appended with AmB and found an IC_50_ of approximately 0.60 ug/mL for all samples in the same cell line. As can be seen, PLA + AmB obtained values almost double those found by the researchers, while PCL + AmB obtained considerably lower values, indicating greater toxicity of these in relation to the macrophages in question.

### 3.3. Zebrafish Embryo–Larvae Toxicity (ZELT) Assessment

**Figure 5:**
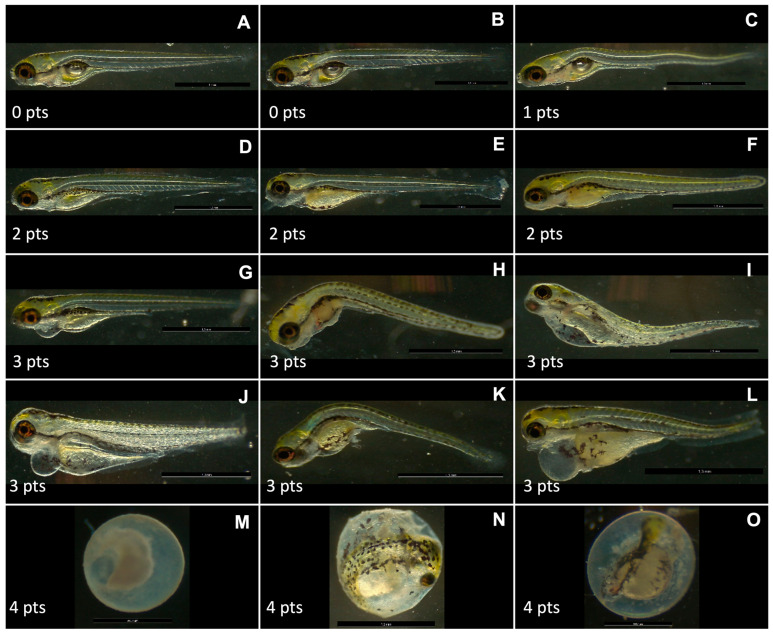
Images exemplifying zebrafish larvae (120 hpf) observed in present study as well as score attributed to each one. Pts: points; hpf: hours post-fertilization. (**A**,**B**) Larvae considered normal in relation to what is expected for 120 hpf, score 0. (**C**) Larvae with a crooked spine, score 1. (**D**–**F**) Larvae with poorly inflated swim bladder and a lot of not consumed yolk, score 2. (**G**) Larvae with underinflated swim bladder, yolk excess, and pericardial edema, score 3. (**H**–**L**) Deformed larvae with a crooked spine, edemas, yolk excess, and underinflated swim bladder, score 3. (**M**) Clot in 24 hpf, score 4. (**N**,**O**) Total of 120 hpf larvae that did not hatch, both with absence of somites, score 4. Images were taken in Leica S9i stereoscope coupled with LAS X software.

Figure 5 shows image examples of 120 hpf zebrafish larvae tested with the PNPs obtained in the present work, as well as the score assigned to each one.

#### 3.3.1. Effects of Non-Loaded PNPs on Zebrafish Embryos

Testing P80 concentrations was also necessary because, at the start of this study, the preparation of PNP did not involve the ultracentrifugation step, meaning that the excess of this water-soluble surfactant was not removed from the final sample. Therefore, since it could interfere with the results to be obtained, it also needed to be tested individually.

As demonstrated in the Supplementary Data, many of the groups tested, including those referring to the concentrations of P80 present in the final dilutions, presented high toxicity scores, differing from the negative control group, which presented a survival rate of 95%.

As reported by Ali et al. (2011), in a study that evaluated the toxicity of P80 using 24 hpf zebrafish embryos, the LC50 obtained was 323.4 µg/mL, an approximate value also used in the present study (307.2 µg/mL), which had a 75% death rate [43]. Therefore, despite the difference in post-fertilization time in which the trials were started, we concluded that adding an ultracentrifugation step to the production of the PNPs was necessary to remove the excess P80 from the samples.

**Figure 6:**
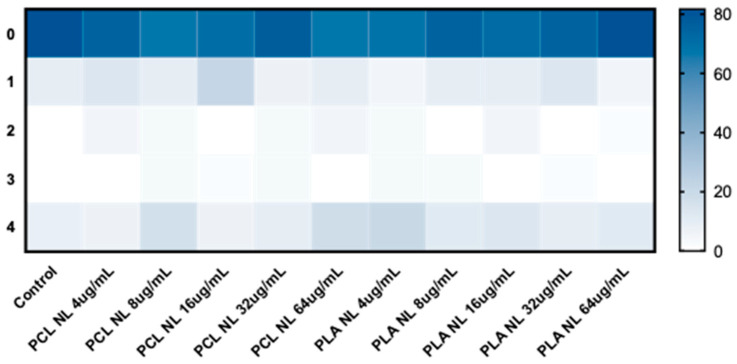
Percentages for each toxicity score obtained in zebrafish embryo toxicity test at 120 h post-fertilization after exposure to NL PNP. PCL: polycaprolactone; PLA: poly(lactic acid); NL: non-loaded; PNP, polymeric nanoparticle. *n* = 20 per replicate (3), *p* > 0.05 in Kruskal–Wallis test followed by Dunn’s multiple comparisons test.

After P80 was removed from the samples, the average score given to non-loaded PNPs (NL-PNP) decreased at all tested concentrations, resulting in no significant difference compared to the negative control group, in which there was an average 8.3% death rate (larvae with a score of 4). Figure 6 shows the obtained percentages for each toxicity score (0–4) for the NL-PNP.

Most larvae obtained a score of zero points for all tested NL-PNP and negative control groups, demonstrating that the polymers used were not the ones causing larvae toxicity or death. Additionally, it is important to clarify that all larvae that received a score different from 4 had hatched.

However, this extra preparation step was not able to reduce the toxicity of AmB-loaded PNPs, since all embryos tested with them, at the firstly studied concentration range, coagulated in less than 3 hpf. This suggests it is likely that the drug’s concentrations, at first round concentrations, were the true cause of death for 100% of the embryos.

**Figure 7:**
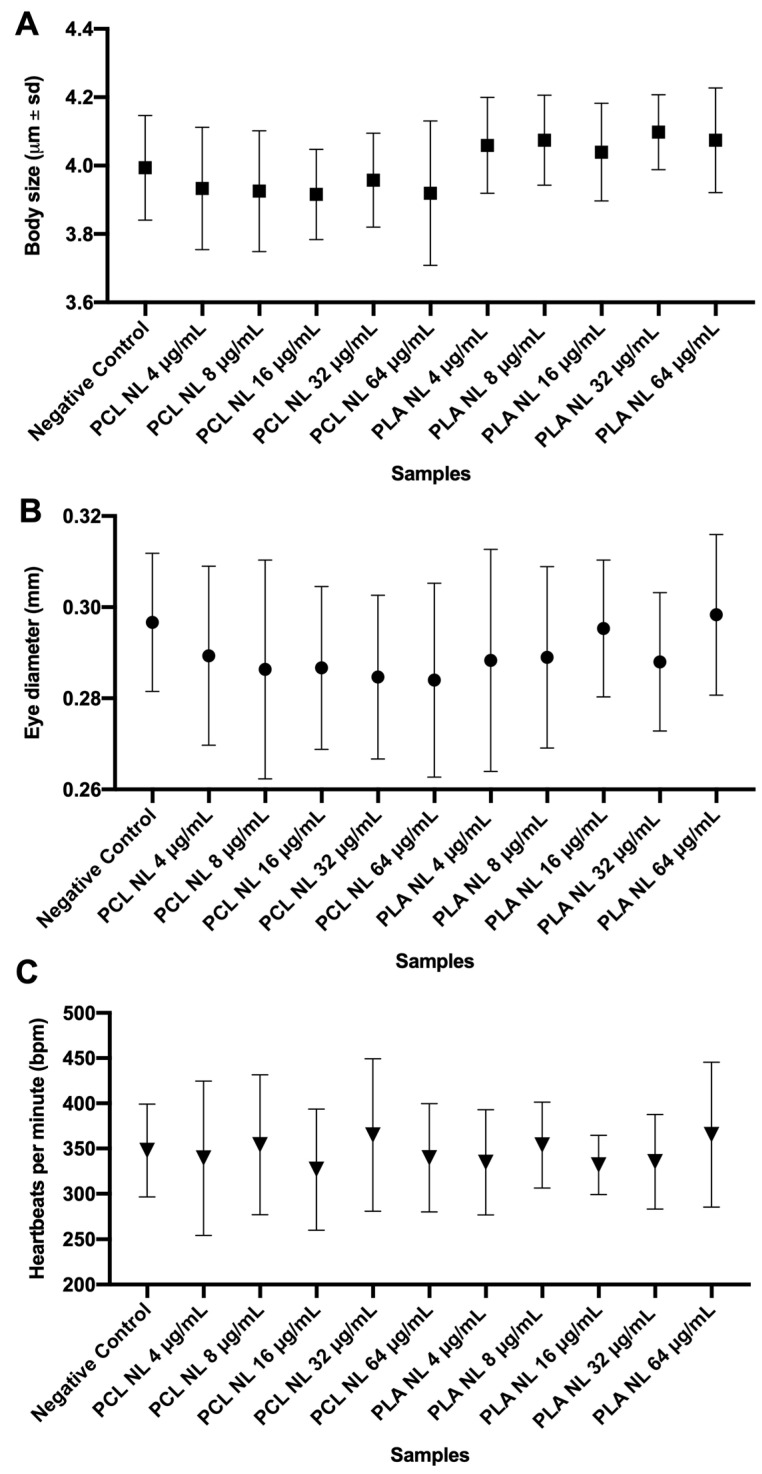
Body size (**A**), eye diameter (**B**), and heartbeats per minute (**C**) analysis of zebrafish larvae at 120 h post-fertilization incubated with polycaprolactone (PCL) or poly(lactic acid) (PLA) non-loaded (NL) nanoparticles. Data shown as mean ± standard deviation. *n* = 10 in each group per replicate (3), *p* > 0.05 in one-way ANOVA test followed by Dunnet’s multiple comparisons test.

Regarding the NL-PNP, the most frequent injuries observed were the absence or low inflation of the swim bladder, an exaggerated amount of yolk at 5 dpf, crooked spine, and pericardial edema. To determine whether NL-PNP affected the embryos’ growth and development, the head-to-tail body size and diameter of the larvae eyes were measured from images obtained on the last day of the test (120 hpf), using Leica’s LAS X software. Figure 7A,B shows that none of the groups differed from the negative control group, indicating that NL-PNP did not impact larval development.

Also, heartbeats were measured to check whether there were changes in the groups tested with NL-PNP compared to the negative control. Changes in the heart rate of zebrafish larvae could correspond to cardiotoxic effects on cardiac function or malformations in the cardiovascular system [44]. However, despite the occasional occurrence of pericardial edema in a few tested animals, none of the groups were different from the negative control group, demonstrating that NL-PNP did not cause essential changes in the heart rate of the studied animals, suggesting them to be safe for the zebrafish larvae heart (Figure 7C).

No opaque regions were found in the evaluated embryos/larvae images in general, which indicates no massive cell death, and therefore suggesting no damage to the organs in question [43,44].

In a PCL PNP biodistributions assay (213.4 ± 27.86 nm) using 7 dpf zebrafish larvae, microinjecting them into the bloodstream, it was demonstrated that PCL PNPs were rapidly sequestered by macrophages, and degraded within about 1 h after injection [45]. This result may help explain the low toxicity found for the PCL NL-PNP studied, as they may be degraded by macrophages, which were present in embryos since 30 hpf [18]. Additionally, it is worth mentioning a 2021 study in which researchers used PCL to nanoencapsulate ascorbic acid and tested these PNPs in zebrafish embryos, also obtaining favorable results regarding their toxicity [46].

Regarding PLA NL-PNP, the results found can be compared to those obtained by Patricio (2016), who studied the toxicity of PLA + PVA NL-PNP (PLA, poly(vinyl alcohol), ranging from 153 ± 58 nm to 273 ± 160 nm, in 3 to 7 dpf zebrafish larvae [25]. Regarding cardiotoxicity, Patricio (2016) identified that PNPs affected the heart rate of larvae in a dose-dependent manner compared to the control group. This result differs from that found in our work, in which no changes in the bpm of 5 dpf larvae were identified. However, a possible explanation for this difference could be the PVA in Patricio’s samples and the time after fertilization when the experiment with the animals started. Regarding morphological changes, in both studies, no differences were found in the toxicity scores of the groups studied compared to the control.

Still, another study demonstrated that PLA can be toxic to zebrafish embryos. The researchers observed increased mortality, reduced hatching rate, decreased body and eye size, and slowed body movement. However, the concentration at which they found such effects was 100 g/mL, well above those studied in the present study [47].

Since cholesterol is the most abundant lipid in the zebrafish embryo [48], we believe that the death generated by AmB was related to two factors: (1) AmB is capable of interacting with cholesterol [49]; (2) the ZELT test, at this round, began approximately one hour after fertilization, is the point in time where the embryo has between 4 and 16 cells [50]. Therefore, due to these factors, it is possible to assume that the damage caused by AmB is irreversible, leading to death within a few hours. This led us to perform a ZELT’s second round, aiming to demonstrate whether the AmB nanoformulation can reduce the toxicity of the free drug in more developed organisms. Therefore, we decided to test their toxicity in (1) lower concentrations and (2) older embryos.

#### 3.3.2. Impacts of AmB-Loaded PNPs and Free Drug on Zebrafish Embryos

**Figure 8:**
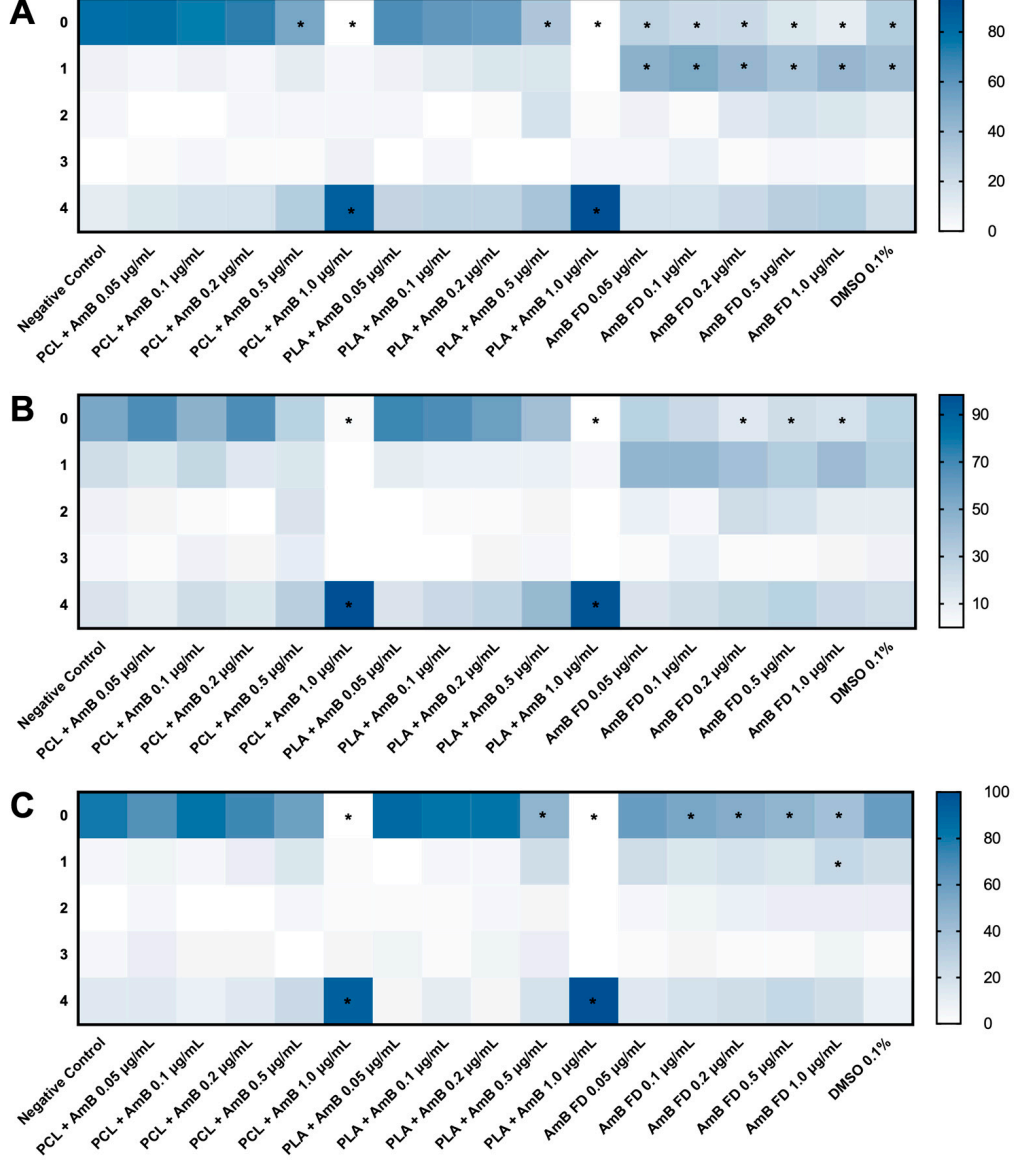
Percentages for each toxicity score (0–4) obtained in zebrafish embryo toxicity test at 120 h post-fertilization after exposure to AmB-loaded PNPs, free drug, or 0.1% DMSO. (**A**) Test started at 1 hpf; (**B**) test started at 6 hpf; and (**C**) test started at 24 hpf. Hpf: hours post-fertilization; AmB: amphotericin B; PCL: polycaprolactone; PLA: poly(lactic acid); FD: free drug; DMSO: dimethylsulfoxide; PNP, polymeric nanoparticle. *n* = 20 per replicate (3), *p* > 0.05 in Kruskal–Wallis test followed by Dunn’s multiple comparisons test. * represents differences between sample and negative control (E3 medium).

Observing the heat maps in Figure 8, it is possible to note that PCL + AmB PNPs were less toxic to zebrafish embryos than PLA + AmB. Regarding FD, although most of the scores assigned were 1 instead of 0, the same occurred in the 0.1% DMSO solvent control, leading us to believe that the toxic effects scored refer to the solvent and not to free AmB. Likewise, although all FD concentrations differed from the negative control in scores 0 and 1, they did not differ from the solvent control.

Another noticeable point is that, in general, the toxicity observed was directly proportional to the concentration tested since the color (heat) intensity assigned to the “0 points” category decreased as the AmB concentration in the medium increased. Finally, it is worth mentioning that, in relation to both PNP + AmB, at a concentration of 1.0 μg/mL, the score 4 percentage was 86.7 ± 23.1% for PCL + AmB and 93.3 ± 11.5% for PLA + AmB, indicating a very high level of lethality.

Next, using the data obtained for the scoring and considering the animals that received a score of 2 or more points as “toxicity present” and those scored with 4 as “dead”, the toxic 50% (TC50) and lethal 50% (LC50) concentrations were calculated, the values obtained for which are shown in Table 3.

**Figure 9:**
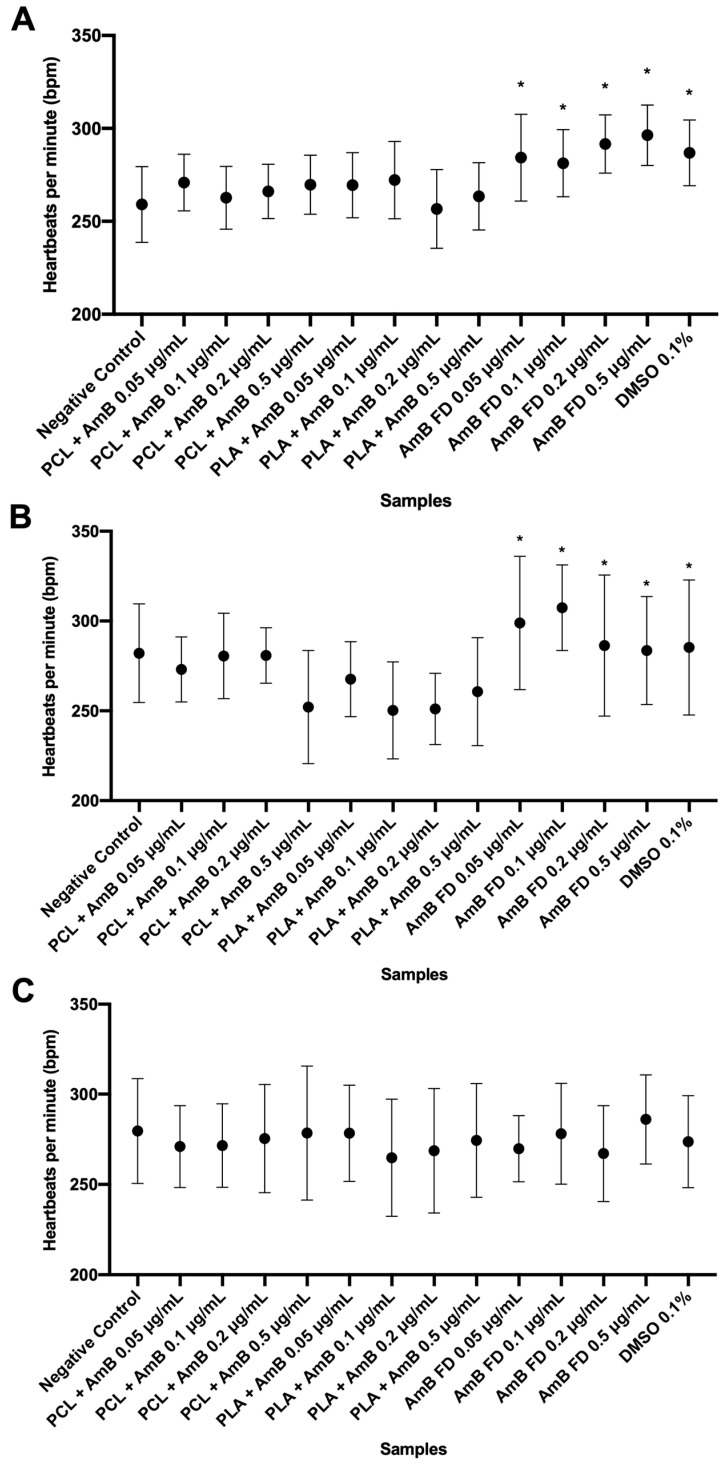
Heartbeats per minute analysis of zebrafish larvae at 120 hpf and incubated with AmB-loaded PNPs or FD. (**A**) Test started at 1 hpf; (**B**) test started at 6 hpf; and (**C**) test started at 24 hpf. Hpf: hours post-fertilization; AmB: amphotericin B; PCL: polycaprolactone; PLA: poly(lactic acid); FD: free drug; DMSO: dimethylsulfoxide; PNP, polymeric nanoparticle. Data shown as mean ± standard deviation. *n* = 10 in each group per replicate (3), *p* > 0.05 in one-way ANOVA test followed by Dunnet’s multiple comparisons test. * represents differences between sample and negative control (E3 medium).

**Figure 10:**
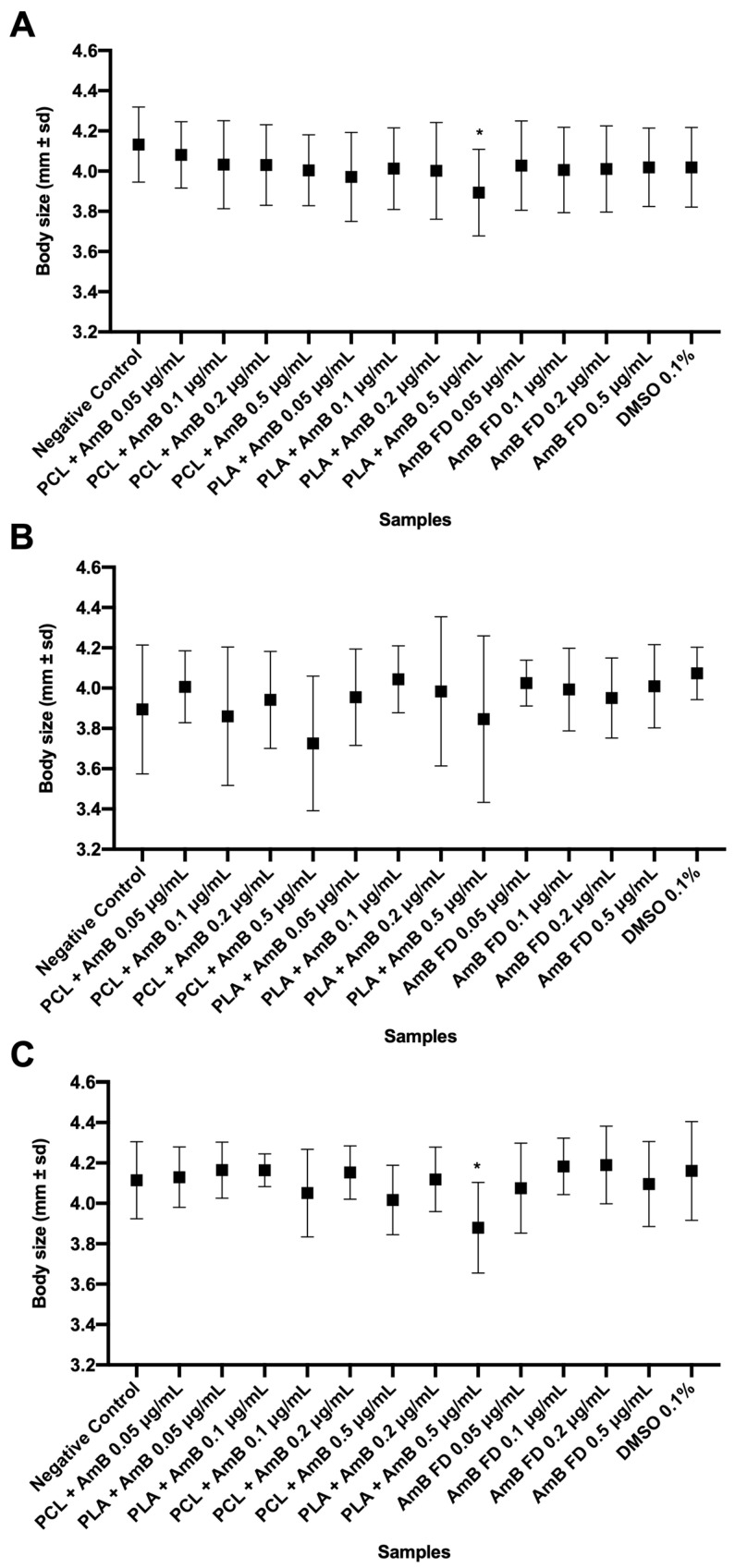
Body size measurement analysis of zebrafish larvae at 120 hpf and incubated with AmB-loaded PNPs or FD. (**A**) Test started at 1 hpf; (**B**) test started at 6 hpf; and (**C**) test started at 24 hpf. Hpf: hours post-fertilization; AmB: amphotericin B; PCL: polycaprolactone; PLA: poly(lactic acid); FD: free drug; DMSO: dimethylsulfoxide; PNP, polymeric nanoparticle. Data shown as mean ± standard deviation. *n* = 10 in each group per replicate (3), *p* > 0.05 in one-way ANOVA test followed by Dunnet’s multiple comparisons test. * represents differences between sample and negative control (E3 medium).

**Figure 11:**
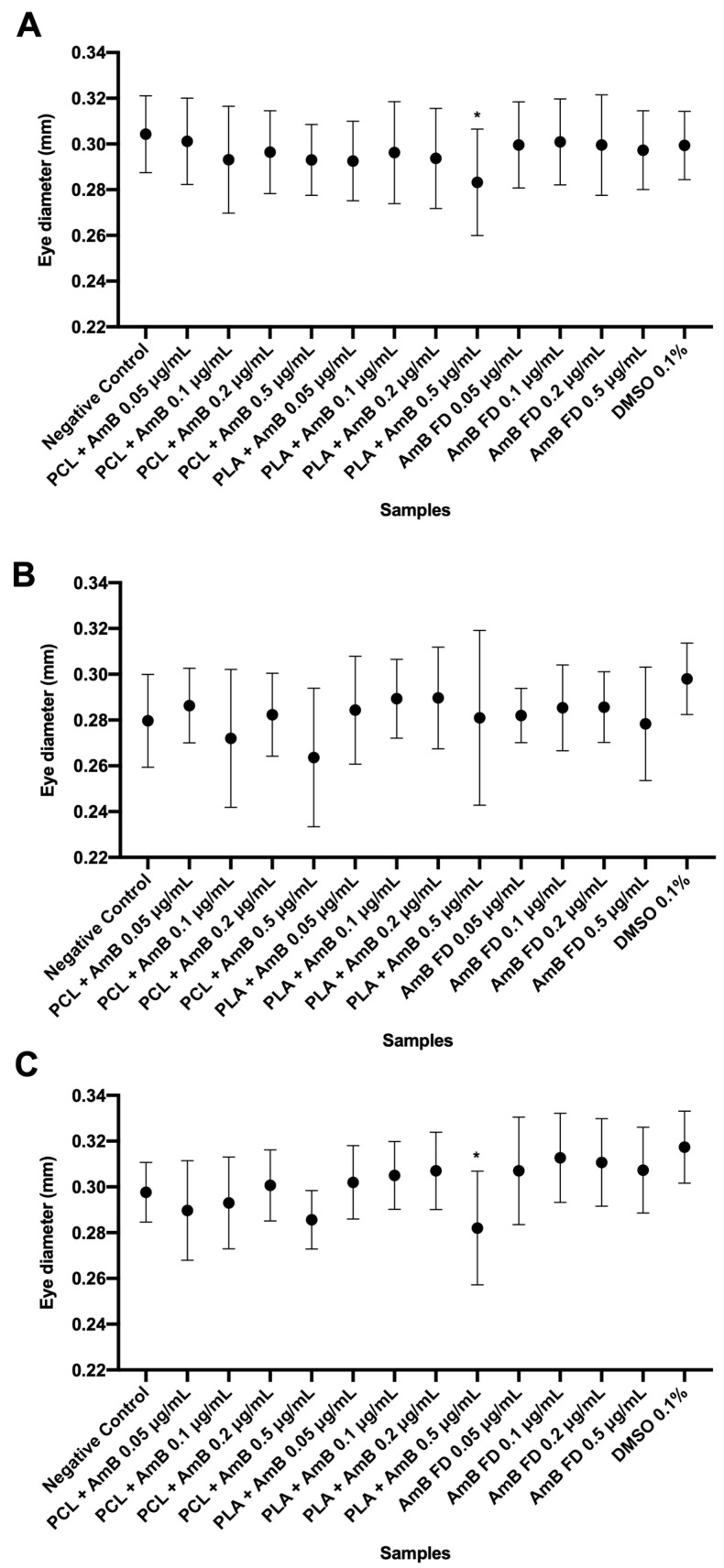
Eye diameter analysis of zebrafish larvae at 120 hpf and incubated with AmB-loaded PNPs or FD. (**A**) Test started at 1 hpf; (**B**) test started at 6 hpf; and (**C**) test started at 24 hpf. Hpf: hours post-fertilization; AmB: amphotericin B; PCL: polycaprolactone; PLA: poly(lactic acid); FD: free drug; DMSO: dimethylsulfoxide; PNP, polymeric nanoparticle. Data shown as mean ± standard deviation. *n* = 10 in each group per replicate (3), *p* > 0.05 in one-way ANOVA test followed by Dunnet’s multiple comparisons test. * represents differences between sample and negative control (E3 medium).

As shown, the concentration of 1.0 μg/mL was lethal for most of the larvae, making it statistically impossible to perform the tests of heart rate, body size, and eye diameter at this concentration. Therefore, the analyses were performed with the other concentrations tested (Figure 9, Figure 10 and Figure 11).

As shown in Figure 9, although FD appears to accelerate the animals’ BPM, in relation to the negative control, the group tested with DMSO also showed such variation. This suggests that, in fact, it is the DMSO that is causing the observed tachycardia and not AmB. This acceleration was not observed for the animals whose testing began at 24 hpf, insinuating that this toxicological finding is time-related.

As observed in Figure 10 and Figure 11, only the group tested with PLA + AmB PNPs at 0.5 mg/mL showed significantly reduced growth in relation to the negative control and the other groups. Observing these data and comparing them to the calculated TC50 and CL50, it is possible to infer that these PNPs are more toxic to zebrafish larvae than PCL + AmB or FD. On the other hand, it can be suggested that, by nanoencapsulating the drug, its penetrability into zebrafish embryos was increased, which can be seen as an improvement regarding the drug’s absorption and bioavailability.

**Figure 12:**
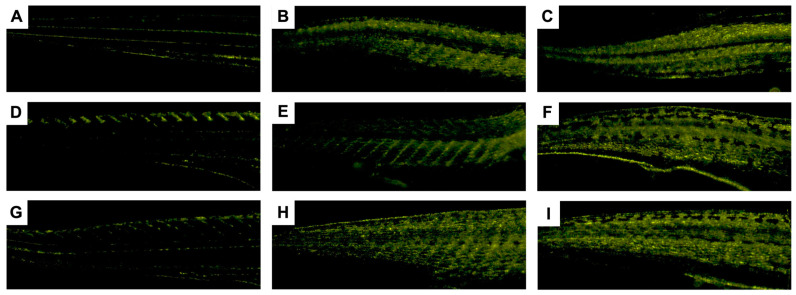
Fluorescence microscopy images of 120 hpf zebrafish larvae tested with NL PNP, PNP + AmB, and AmB FD. (**A**) Negative control (E3 medium); (**B**) DMSO 0.1%; (**C**) AmB FD 1.0 mg/mL; (**D**) PCL NL; (**E**) PCL + AmB 0.5 mg/mL; (**F**) PCL + AmB 1.0 mg/mL; (**G**) PLA NL; (**H**) PLA + AmB 0.5 mg/mL; (**I**) PLA + AmB 1.0 mg/mL. Hpf: hours post-fertilization; NL: non-loaded; AmB: amphotericin B; PCL: polycaprolactone; PLA: poly(lactic acid); FD: free drug; DMSO: dimethylsulfoxide; PNP, polymeric nanoparticle.

To test this theory, knowing that AmB is capable of emitting fluorescence in the 560 nm region [51], we decided to use fluorescence microscopy to identify whether, after 120 h of treatment with PCL + AmB, PLA + AmB, or AmB FD, the zebrafish larvae emitted fluorescence above the basal level, as verified through the negative control. The images obtained are shown in Figure 12.

As can be seen in Figure 12, the fluorescence intensity increased for the larvae tested with both PNP + AmB, mainly in the caudal portion of the animals, especially PLA + AmB. As seen in Figure 12E, the PCL + AmB PNPs, at a concentration of 0.5 mg/mL, fluoresced slightly less than that of PLA + AmB at the same concentration (Figure 12H), indicating a greater penetration power of the latter. This result is in line with what was observed previously, in relation to LC_50_ and the development of the body and eyes analyses. At the same time, it was also possible to observe this fluorescence increase in those tested with AmB FD (Figure 12C). However, the group that received the DMSO 0.1% solvent control—the same concentration in which AmB FD is dissolved—also fluoresced, indicating the possibility of the observed fluorescence being caused by the solvent (Figure 12B). In any case, it is known that DMSO acts as a penetrability facilitating agent [52], which would help AmB penetrate the larvae’s body. We also tested non-loaded PNPs to ensure that the observed fluorescence was not actually caused by the polymers. As can be seen in Figure 12D,G, the larvae tested with PNP NL fluoresced as little as the negative control, not demonstrating fluorescence levels above baseline. Therefore, these results lead us to believe that PNP + AmB actually penetrated the body of the tested larvae, spreading throughout their entire body.

In a 2010 published work [53], Wang et al. tested pure AmB and a newly synthesized derivative in zebrafish embryos. However, they started the test with embryos that were at 6 hpf and, at this point in time, they are already in the gastrula phase of development, having more than a thousand cells and being in a shield format [50]. Even so, the researchers found an LC_50_ value of 0.09 mg/mL for AmB [53].

However, as our results show, changing the start time of ZELT in a few hours can influence obtained results, especially when comparing toxic and lethal concentrations obtained at 1, 6, and 24 hpf. It is noticeable that, as the starting time advances, the animals become less susceptible to the toxic effects of the samples. A possible explanation is the constant increase in the number of cells present in the embryos at these two moments. While at 6 hpf, they already have more than a thousand cells, at 24 hpf, they are quite developed, with several organs being formed, and it is already possible to identify eyes, yolk sac, somites, among others [50]. Thus, the impact caused by AmB on the animal is reduced, which, as previously discussed, is possibly generated by its ability to damage cell membranes due to interactions with cholesterol and therefore with fewer cells, a higher damage to the animals can be observed.

Additionally, AmB tends to form auto-aggregates in aqueous environments due to its low solubility [54]. In fact, Fernandez-Garcia et al. (2022) demonstrated that its oligomeric state appears to be the most effective against fungal infections by binding to the ergosterol present in their cell membranes [31]. This oligomeric state was also described in the literature as a “sponge”, which acts by sequestering the membrane lipids more efficiently than AmB’s monomeric state [55,56].

The hypsochromic change observed in Figure 3 may be indicative of the interaction between the polymers and AmB molecules, as observed by Das and Devarajan (2020), in a study where they prepared AmB-loaded nanoparticles with the Gantrez polymer [57]. In this article, the authors suggest that these interactions provide thermodynamic stability advantages, making PNPs act as reservoirs of the drug, releasing it into the surrounding medium over time and increasing its efficacy. This finding may explain the toxicity observed in PNP + AmB and its absence in AmB FD, which, despite also appearing to be in an oligomeric state in Figure 3, caused less of an effect on zebrafish larvae and tested cell lines.

In the study by Rossi et al. (2020) [58], AmB was tested alone and in combination with several other drugs to identify whether they would be more potent as an antifungal agent in a zebrafish larvae model infected with C. albicans. A concentration of 0.03 mg/mL of AmB was applied for all combinations. In the specific case of the test performed with pure AmB, the researchers obtained no embryo mortality over the 96 h of testing. However, this concentration is below the range used in the present study, where low mortality was also observed. Still, in agreement with the results demonstrated here, the researchers also observed that, in some cases, greater toxicity was identified in zebrafish embryos than in cell tests. They then suggest that the differences may be related to the fact that the zebrafish embryo is a complex organism in which drugs generally have different toxicokinetics than in cultured cells and may already undergo some metabolism. Furthermore, at less than 72 hpf, they have already developed some of their main organs, such as the heart, liver, and nervous system, and these can be affected by the drugs.

In another study, published in 2022, researchers tested pure AmB and inserted the drug into medium-chain length polyhydroxyalkanoate biopolymer microspheres [59]. Regarding the free drug, they obtained results that were very similar to those found in the present study. Although they did not calculate LC_50_ and TC_50_, it is noted that approximately 50% of the embryos showed signs of toxicity around 0.7–0.8 mg/mL and that at 2 mg/mL, 100% lethality already occurred. On the other hand, for the microspheres containing AmB, their results demonstrated a 10x reduction in toxicity, making it possible to reach concentrations such as 100 μg/mL without apparent toxic signs in 120 hpf larvae. However, these structures presented 7 to 8 μm of diameter, 50× larger than the PNPs studied in our work, and possibly faced some difficulty penetrating the embryo chorion since the pores found in this protective layer vary between 400 and 700 nm in diameter [60,61,62].

## 4. Conclusions

This work aimed to produce, characterize, and study the toxic potential of polymeric nanoparticles containing amphotericin B by MTT assays and zebrafish embryo–larvae toxicity tests. Our initial findings pointed out that polysorbate 80 needed to be removed from the supernatant at the end of PNP preparation, since it showed high toxicity to zebrafish embryos between 1 and 120 hpf.

Next, regarding the safety of the nanostructures used, it can be concluded that they proved to be safe in the test with 1–120 hpf zebrafish embryos since they did not show differences in relation to the control group at any of the concentrations tested and in none of the evaluated parameters.

Finally, in the toxicological tests, PNPs containing AmB were shown to be more toxic than FD, both in vitro and in vivo, probably due to AmB FD aggregates forming in aqueous medium. However, this observed intensification in toxicity also suggests an increase in its absorption, a characteristic desired to overcome the drug’s bioavailability barrier. Therefore, even though the toxicological results indicate an increase in toxicity with AmB nanoencapsulation, we still believe that the future clinical usage of these PNPs can be interesting in a dosage-reducing manner.

## Figures and Tables

**Table 1:** Results of size (nm), polydispersity index, and zeta potential (mV) analysis of recovered polymeric nanoparticles non-loaded and loaded with amphotericin B.

	PNP NL			PNPs Loaded with AmB					
	Size (nm)	PdI	Zeta Potential (mV)	Size (nm)	PdI	Zeta Potential (mV)	[AmB] (mg/mL)	EE (%)	DL (%)
PCL	114.6 ± 10.2	0.126 ± 0.021	−29.0 ± 7.6	163.4 ± 1.8	0.142 ± 0.033	−24.6 ± 3.0	160.50 ± 8.14	21.66 ± 10.04	5.41 ± 2.51
PLA	171.9 ± 5.9	0.118 ± 0.018	−31.8 ± 9.6	202.4 ± 16.1	0.114 ± 0.030	−28.2 ± 8.5	165.64 ± 9.43	27.15 ± 14.96	6.79 ± 3.74

PdI: polydispersity index; PLA: poly(lactic acid); PCL: polycaprolactone; PNP: polymeric nanoparticle; NL: non-loaded; AmB: amphotericin B; EE: entrapment efficiency; DL: drug loading. Analysis carried out at 25 °C with He-Ne laser (λ = 633 nm) and fixed-angle detector at 90°. Nanoparticles were dispersed in E3 medium (5 mM NaCl; 0.17 mM KCl; 0.33 mM CaCl_2_; 0.33 mM MgSO_4_). Results are expressed as mean ± standard deviation, with *n* = 4 for each group.

**Table 2:** IC_50_ values obtained in MTT assay using HEK293, HepG2, and J774.A1 cell lines, tested with non-loaded or AmB-loaded PNP or FD.

	PLA NL PNP	PCL NL PNP	PLA + AmB PNP	PCL + AmB PNP	AmB FD
HEK293	4.775 μg/mL	7.839 μg/mL	3.759 μg/mL	6.758 μg/mL	>10 μg/mL
HepG2	>10 μg/mL	>10 μg/mL	>10 μg/mL	>10 μg/mL	>10 μg/mL
J774 A1	>10 μg/mL	1.082 μg/mL	1.633 μg/mL	0.2406 μg/mL	1462 μg/mL

IC_50_, inhibitory concentration; AmB: amphotericin B; PCL: polycaprolactone; PLA: poly(lactic acid); NL: non-loaded; FD: free drug; PNP, polymeric nanoparticle.

**Table 3:** TC50 and LC50 obtained in zebrafish embryo–larvae toxicity test started at 1, 6, and 24 hpf of AmB-loaded PLA or PCL PNPs or free drug.

		PLA + AmB PNP	PCL + AmB PNP	AmB FD
1 hpf	TC_50_	0.1536 μg/mL	0.3204 μg/mL	1.034 μg/mL
	LC_50_	0.3982 μg/mL	0.5575 μg/mL	<1.5 μg/mL
6 hpf	TC_50_	0.3497 μg/mL	0.3940 μg/mL	<1.5 μg/mL
	LC_50_	0.4040 μg/mL	0.5776 μg/mL	<1.5 μg/mL
24 hpf	TC_50_	0.5454 μg/mL	0.5582 μg/mL	<1.5 μg/mL
	LC_50_	0.5584 μg/mL	0.6254 μg/mL	<1.5 μg/mL

TC_50_, toxic concentration 50%; LC_50_, lethal concentration 50%; Hpf: hours post-fertilization; AmB: amphotericin B; PCL: polycaprolactone; PLA: poly(lactic acid); FD: free drug; PNP, polymeric nanoparticle.

## Data Availability

Data are available upon request.

## Supplementary Materials

The following supporting information can be downloaded at: https://www.mdpi.com/article/10.3390/pharmaceutics17010116/s1, Table S1: Relative frequency, in percentage, of attributed scores to zebrafish embryos tested with non-ultracentrifuged PNP samples, at 120-h post-fertilization.
