# Supplementary material for: Amphotericin B Encapsulation in Polymeric Nanoparticles: Toxicity Insights via Cells and Zebrafish Embryo Testing
Source: Pharmaceutics. 2025 Jan 16;17(1):116. doi: 10.3390/pharmaceutics17010116 (PMC11768399; doi:10.3390/pharmaceutics17010116)
Supplement: Supplementary file 1 [file pharmaceutics-17-00116-s001.zip › pharmaceutics-3346339-supplementary.pdf]

pharmaceutics MDPI

Supplementary Data

Non-ultracentrifuged PNP zebrafish embryo toxicity test.

The embryos tested with PNP loaded with AmB (PNP+AmB) showed 100% death of all embryos within approximately three hours after starting the test. The non-loaded PNP groups presented death and/or malformations such as absence of somites, pericardial edema, developmental delay, and lack of swim bladder inflation. However, the groups tested with P80 alone also showed toxicity levels above that of the control group. Therefore, it was not possible to determine, at this point, whether the non-loaded PNP (NL-PNP) were causing the toxic effects.

Table S1: Relative frequency, in percentage, of attributed scores to zebrafish embryos tested with non-ultracentrifuged PNP samples, at 120-h post-fertilization.

**Groups**	**Scoring Frequency (%)**				
	**0**	**1**	**2**	**3**	**4**
Negative Control	85	10	-	-	5
PLA NL 4 µg/mL	60	10	5	5	20
PLA NL 8 µg/mL	65	10	-	-	25
**PLA NL 16 µg/mL***	60	15	-	10	15
**PLA NL 32 µg/mL***	15	15	5	15	50
**PLA NL 64 µg/mL***	-	-	-	-	100
**PLA+AmB 4 µg/mL***	-	-	-	-	100
**PLA+AmB 8 µg/mL***	-	-	-	-	100
**PLA+AmB 16 µg/mL***	-	-	-	-	100
**PLA+AmB 32 µg/mL***	-	-	-	-	100
**PLA+AmB 64 µg/mL***	-	-	-	-	100
PCL NL 4 µg/mL	85	10	-	-	5
PCL NL 8 µg/mL	65	5	0	-	30
**PCL NL 16 µg/mL***	-	10	5	20	65
**PCL NL 32 µg/mL***	-	-	-	20	80
**PCL NL 64 µg/mL***	-	-	-	5	95
**PCL+AmB 4 µg/mL***	-	-	-	-	100
**PCL+AmB 8 µg/mL***	-	-	-	-	100
**PCL+AmB 16 µg/mL***	-	-	-	-	100
**PCL+AmB 32 µg/mL***	-	-	-	-	100
**PCL+AmB 64 µg/mL***	-	-	-	-	100
P80 38.4 µg/mL	35	35	5	5	20
**P80 76.8 µg/mL***	5	45	-	-	50
**P80 153.6 µg/mL***	-	-	-	10	90
**P80 307.2 µg/mL***	-	-	-	25	75
**P80 614.4 µg/mL***	-	-	10	5	85

PNP: polymeric nanoparticles; PLA: poly(lactic acid); PCL: polycaprolactone; AmB: Amphotericin B; P80: polysorbate 80. Results are expressed as percentage. *Statistically different from the control group ($p < 0.05$) in the Kruskal-Wallis test followed by Dunn's multiple comparisons test (n = 20 per experimental group).
